# Aortic valve replacement in a patient with sickle cell disease—Are we justified to perform surgery in the TAVI era?

**DOI:** 10.1002/ccr3.4085

**Published:** 2021-03-29

**Authors:** Ramanish Ravishankar, Leenah Turkistani, Yasser Elghoneimy, Imthiaz Manoly

**Affiliations:** ^1^ University of Edinburgh Edinburgh UK; ^2^ Imam Abdulrahman Bin Faisal University Dammam Saudi Arabia; ^3^ Department of Cardiothoracic Surgery James Cook University Hospital Middlebrough UK

**Keywords:** cardiopulmonary bypass, cardiothoracic surgery, hemotology, sickle cell disease

## Abstract

Not all variants of SCD need the same management and this should be decided on a case‐by‐case basis. Heterozygous SCD patients can undergo cardiac surgery without the need for intraoperative exchange transfusions with good clinical outcomes.

## Graphical abstract

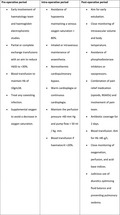

## 1 INTRODUCTION

Sickle cell disease (SCD) denotes a spectrum of genetic disorders due to the hemoglobin mutation characterized genotypically. This syndrome is conceived with the replacement of one of the beta‐globin subunits in hemoglobin A with hemoglobin S (HbS).^1^ The severity and prognosis of this condition is determined by the presence of another allele which can then categorize SCD into a homozygous or heterozygous sickle cell trait.^1^ As an autosomal recessive hemoglobinopathy, SCD is prevalent typically in the African, Southern European, Indian, and isolated pockets of the Mediterranean population.^2^

Sickle cell disease is known to cause significant mortality due to hemolytic anemia, and morbidity due to its involvement in the infarction of major organs including spleen and bone and major organ systems resulting in stroke, cardiovascular complications, and pulmonary hypertension.^3^

Sickling or a vaso‐occlusive crisis is usually triggered by various factors such as hypoxemia, hypothermia, or dehydration.^3^ These triggering factors are quite common in cardiac surgery, involving cardiopulmonary bypass (CPB), which can result in fatal sickle cell‐related complications. As a result, it is imperative to choose the right strategy to minimize mortality and morbidity in patients with SCD.

## 2 CASE SUMMARY

A 50‐year‐old female from Eastern province of Saudi Arabia presented with symptomatic severe aortic stenosis which was deemed NYHA Class III. Her main symptom was shortness of breath.

She was known to have a sickle cell trait (HbAS), without any previous reported vaso‐occlusive crisis. A transthoracic echocardiogram (TTE) demonstrated severe aortic stenosis and moderate LV systolic dysfunction. Her coronaries were normal on angiogram; hence, surgical aortic valve replacement was recommended. The patient however, having heard about transcatheter aortic valve replacement (TAVI), wanted to know if cardiac surgery would increase her mortality and poor prognosis. She also wanted to be informed of any alternative, particularly TAVI. Further to the literature review and the multidisciplinary team meeting, she was reassured and subsequently consented for surgical biological aortic valve replacement. The tissue valve was suggested in anticipation of avoiding long‐term anticoagulation and further potential bleeding complications in this cohort.

## 3 INVESTIGATIONS

As this patient had a known heterozygous sickle cell trait, care was taken to determine the baseline characteristics of her hemoglobin. Aside from a complete blood count to rule out anemia, decreased hematocrit, thrombocytosis and increased reticulocyte count, a peripheral blood smear was also taken in addition to electrophoresis.

Pulmonary function tests were undertaken to identify any underlying respiratory compromise such as atelectasis due to her sickle cell trait. Assessing the baseline respiratory function can also help target post‐op recovery and aid any anesthetic decisions. Furthermore, a transcranial Doppler ultrasound was undertaken to assess any risk of stroke, a complication which has a higher prevalence in those with sickle cell anemia. However, the limitation to this being that the Doppler is not a very specific investigation to identify this. A chest X‐ray was performed to assess for any pulmonary consolidation or opacity, signs which could suggest an acute chest syndrome (ACS) as a consequence of sickle cell anemia. The patient also had their renal and liver function checked in order to assess for any underlying chronic pathology such as sickle cell nephropathy and hepatopathy, presentations of such which are very common in HbS patients. The renal function was normal which allowed the patient to undergo surgery under cardiopulmonary bypass.

As with any patient suffering from severe aortic stenosis, a TTE was used to grade the severity of the aortic stenosis. It also allowed for assessment of left ventricular diastolic dysfunction and allowed the team to rule out pulmonary hypertension and tricuspid regurgitation.

## 4 TREATMENT

The patient underwent a biological aortic valve replacement using cardiopulmonary bypass (CPB). Special precautions were taken pre‐, intra‐ and postoperatively in order to minimize mortality and morbidity risk factors. Overall, our objective was to minimize the precipitating factors, optimize the oxygen carrying capacity of hemoglobin and, as a result, avoid complications.

Preoperatively, the patient was admitted early and was involved with the multidisciplinary team including the hematologist, anesthetist, and cardiologist. The patient was optimally hydrated the day before surgery while keeping in mind of severe aortic stenosis. All routine blood work was completed and recorded. Special care was taken to identify the blood group and further cross matching in anticipation of exchange transfusion. The pain team was involved and the patient's tolerance and threshold for pain was documented. As the patient was known to be heterozygous SCD, the decision was made to reserve exchange transfusion for the intraoperative period after further discussion with the hematologist. Incentive spirometry was encouraged to improve the compliance and reduce postoperative atelectasis.

Intraoperatively, the patient had normothermic hydration prior to induction of anesthesia. All intravenous fluids were warmed prior to infusion to achieve this. After establishing arterial and central venous lines, the analgesics were administered as intravenous fentanyl for induction and further boluses during the procedure. Epidural morphine stat and intravenous morphine were administered intermittently. Throughout the procedure, the goal was to maintain a Hb of 10 g/dL. The patient did not require exchange transfusion as we were able to maintain the hemoglobin level. Our strategy was to transfuse packed RBCs instead of exchange transfusion and to reserve the latter if the Hb was >10g/dL. If this were to be the case, exchange transfusion would be undertaken with phenotypically matched blood to prevent alloimmunization. The patient came off cardiopulmonary bypass safely and heparin was reversed with no adverse events. She was subsequently transferred to intensive care in a stable condition.

Postoperatively, the patient was extubated as per the ICU protocol. Supplemental oxygen was administered for the first day irrespective of the normal oxygen saturation and the goal was to maintain an oxygen saturation of >95%. Patient was hydrated generously with hypotonic solutions (5% Dextrose) and aggressive pain management was implemented to avoid any vaso‐occlusive crisis. Incentive spirometry and early ambulation was encouraged and, as a result, the patient had normal postoperative recovery and was discharged on day 6. She had regular follow‐up with Cardiology and Hematology.

## 5 DISCUSSION

Approximately 12 000‐15 000 people live with sickle cell disease in the UK and, with those affected mostly of African or African‐Caribbean origin, this is a rare patient cohort to undergo cardiac surgery in the UK.^4^ As a result, the clinical guidelines for undertaking high‐risk surgery in HbS patients are recommendations with no current RCTs run around the subject. Current guidelines by the British Society of Haematology (BSH) recommend preoperative transfusion for high‐risk (Grade 1C) surgery while maintaining oxygenation, warmth, hydration, adequate anesthetic and surgical technique.^5^ Furthermore, maintaining a HbS concentration <30% has been shown to prevent recurrent ischemic stroke.^5^

However, the general debate between TAVI and SAVR has been prevalent, with the consensus of resorting to TAVI in older patients and those not suitable for an open operation. Lack of long‐term outcomes in TAVI concluded that SAVR is generally favorable in such situations.^6^ However, as the surgical option would be utilizing CPB in our case, the literature was reviewed to assess the clinical efficacy of this.

Yousfzai et al^7^ reported the largest retrospective observational study to date on sickle cell patients who underwent cardiac surgery. They reported consistent and successful outcomes by avoiding intraoperative hypoxia, hypothermia, and vaso‐constrictive agents as recommended by the BSH guidelines.^5^ Their group minimized HbS levels with preoperative and/or perioperative exchange transfusion. Although there was almost an equal share of the pediatric population in their study, they reported only one 30‐day‐mortality and their 4‐year survival rate was 66%. The complications were mainly related to bleeding (6.4%), Stroke (4.3%), Renal (4.3%), and prolonged ventilation (2.4%). This was similarly emulated by Moutaouekkil et al, by presenting two cases where mitral valve repair was preceded by partial exchange blood transfusions.^8^ No adverse outcomes were reported, and an average hospital stay of 10 days.

In addition, Edwin et al,^9^ reported similar outcomes in their study comparing seven patients with HbSS, twenty‐one patients with HbAS and five patients with HbSC. With all patients undergoing cardiac surgery, preoperative exchange transfusion was not used routinely in their case group. Their matched pair case‐control study reported no mortality, or any sickle cell‐related fatal complications. However, the HbSS group significantly required more blood transfusion and a greater length of hospital stay.

By adopting modifications in their CPB strategy including 30% exchange transfusion, continuous hemofiltration during CPB, maintaining Hb levels at 6‐8.5 g/dL, and maintaining a hematocrit of 18%‐28%, Ebrahim et al demonstrated safe conduct of longer operations.^10^ Relevant examples in his study included double valve replacements in homozygous SCD patients with no mortality or sickle cell‐related complications.

Harban et al^11^ from Great Ormond Street hospital reported similar positive results in the pediatric SCD population who underwent congenital cardiac surgery with CPB. All cases proceeded without complication and good surgical results were achieved by lowering the hemoglobin S levels throughout the procedures.

In contrast, Crawford et al,^3^ presented a recent literature review on managing sickle cell disease for such situations. Variable opinions were identified among some clinicians with regards to reducing HbS to <30% through exchange transfusions as this may subsequently lead to large volume transfusion and coagulopathy. The debate between exchange transfusions and simple transfusions have different studies supporting either conclusion. Bocchieri et al,^12^ proposed intraoperative exchange transfusions to reduce the HbS to <5% as a method to mitigate cardiovascular stress and further coagulopathy. However, anecdotal accounts on the other end of the spectrum clarify that simply preventing hypoxia, hypovolemia and acidosis can alleviate the need for exchange transfusion.^13^ This was mirrored in a case series by Frimpong‐Boateng et al; two patients underwent mitral valve replacement with no pre‐ or intraoperative exchange transfusion.^14^ Although both subjects were pediatric patients, they were weaned off CPB with no sickling crisis or blood transfusions after a 1‐year follow‐up.

As a result, our case demonstrates the value of multidisciplinary advice in assessing and treating this patient. Although most studies aforementioned have used exchange or simple transfusion preoperatively as a pre‐emptive act,^3^, ^7^, ^9^ our strategy opted for an exchange transfusion intraoperatively only if the Hb >10g/dL. Furthermore, our treatment plan allowed us to maintain normothermic temperatures by warming intravenous fluids and the addition of supplemental oxygen prevented hypoxia and any subsequent atelectasis. As this patient has the heterozygous sickle cell trait with no history of a vaso‐occlusive crisis and normal pulmonary, renal, hepatic, and cardiac functional tests, we have shown that this strategy is effective for such patients.

In conclusion, experienced centers adopted strategies to prevent SCD‐related crisis which included decreasing the HbS concentration, increasing the hematocrit, increasing the oxygen saturation and maintaining the pH and thereby preventing the acidosis.^3^, ^15^ This review also included the pediatric population in three of the reported studies. With reduction of HbS concentration and avoidance of precipitating factors, cardiac surgery involving CPB can be performed for such patients with acceptable outcomes. The important principles of management of such patients are however to avoid the sickling triggers including hypothermia, hypoperfusion, acidosis, and hypoxia during intracardiac surgeries, which consequently allow a successful recovery. A summary of learning points is presented in Table 1.

**TABLE 1:** Summary of learning points

Preoperative period	Intraoperative period	Postoperative period
Early involvement of hematology team and hemoglobin electrophoretic studies. Partial or complete exchange transfusions with an aim to reduce HbSS to <30%. Blood transfusion to maintain Hb of 10 gm/dL Treat any coexisting infection. Supplemental oxygen to avoid a decrease in oxygen saturation.	Avoidance of hypoxemia maintaining a venous oxygen saturation >80%. Inhaled or intravenous maintenance of anesthesia. Normothermic cardiopulmonary bypass. Warm cardioplegia or continuous cardioplegia. Maintain the perfusion pressure >60 mm Hg and pump flow >50 ml/Kg. min. Blood transfusion if hematocrit <20%.	Aim for early extubation. Close monitoring of intravascular volume and body temperature. Avoidance of phosphodiesterase inhibitors or vasopressors. Combination of pain relief medication (opioids, NSAIDs) and involvement of pain team. Antibiotic coverage for 2 d. Blood transfusion: Aim for Hb >85 g/L. Close monitoring of oxygenation, perfusion, and acid‐base indices. Judicious use of diuretics optimizing fluid balance and preventing pulmonary edema.

## CONFLICT OF INTEREST

None declared.

## AUTHOR CONTRIBUTIONS

RR: involved in writing report. LT: assisted in writing report. YE: contributed key details and YG: cared for the patient. IM: involved in supervising, performing literature review, and writing report.

## ETHICAL APPROVAL

The patient was consented prior to write‐up.

## Data Availability

Data sharing is not applicable to this article as no datasets were generated or analyzed during the current study.
